# The Impact of Anemia on Child Mortality: An Updated Review

**DOI:** 10.3390/nu6125915

**Published:** 2014-12-22

**Authors:** Samuel P. Scott, Lenis P. Chen-Edinboro, Laura E. Caulfield, Laura E. Murray-Kolb

**Affiliations:** 1 Department of Nutritional Sciences, The Pennsylvania State University, University Park, PA 16802, USA; E-Mail: sps5032@psu.edu; 2 Department of Mental Health, Johns Hopkins Bloomberg School of Public Health, Baltimore, MD 21205, USA; E-Mail: lchen78@jhu.edu; 3 Program in Human Nutrition, Department of International Health, Johns Hopkins Bloomberg School of Public Health, Baltimore, MD 21205, USA; E-Mail: lcaulfi1@jhu.edu

**Keywords:** anemia, iron deficiency, child mortality

## Abstract

Iron deficiency anemia and child mortality are public health problems requiring urgent attention. However, the degree to which iron deficiency anemia contributes to child mortality is unknown. Here, we utilized an exhaustive article search and screening process to identify articles containing both anemia and mortality data for children aged 28 days to 12 years. We then estimated the reduction in risk of mortality associated with a 1-g/dL increase in hemoglobin (Hb). Our meta-analysis of nearly 12,000 children from six African countries revealed a combined odds ratio of 0.76 (0.62–0.93), indicating that for each 1-g/dL increase in Hb, the risk of death falls by 24%. The feasibility of a 1-g/dL increase in Hb has been demonstrated via simple iron supplementation strategies. Our finding suggests that ~1.8 million deaths in children aged 28 days to five years could be avoided each year by increasing Hb in these children by 1 g/dL.

## 1. Introduction

Twenty percent of all global deaths occur in children under five years of age [1]. Though impressive progress has been made toward achieving Millennium Development Goal 4 (MDG4) [2]—to reduce the under-five mortality rate (U5MR) by two-thirds from 1990 to 2015—the target will not be reached in developing regions. From 1990 to 2012, the global U5MR declined by 47%, from 90 to 48 deaths per 1000 live births [3]. While developed regions are on track, reaching MDG4 in developing regions would require an average annual rate of reduction of 14.2% from 2011 to 2015 [3]. The reduction of mortality in children, particularly in Sub-Saharan Africa, South and Southeast Asia, is a high public health priority.

From 2000 to 2010 [4], in children aged 1 month–5 years, pneumonia, diarrhea and malaria claimed the most lives. Anemia, the inability of erythrocytes to provide adequate oxygen to the body’s tissues, is also recognized as a direct cause of death when it is severe [5]. Additionally, it is recognized that mild and moderate anemia may also contribute to mortality risk [6]. The focus of this paper is to provide updated estimates on the nature and magnitude of this risk relation; that is, how the risk of death changes as the hemoglobin concentration (Hb) increases in children with mild or moderate anemia.

Globally, 43% of young children (6–59 months) [7] and 25% of older children (5–15 years) [8], a combined 600 million, are estimated to be anemic. The WHO currently defines anemia as having a Hb concentration below 110 g/L in children 6–59 months, below 115 in children 5–11 years and below 120 g/L in children 12–14 years [9]. These thresholds are set at the fifth percentile of the Hb of a normal population [10]. It has been estimated that about half of anemia is due to iron deficiency (ID) [5], with other nutritional deficiencies (folate, vitamin B12 and vitamin A), inflammation and infection and inherited disorders affecting erythrocytes, such as thalassemia, also contributing [11]. The relation between anemia due specifically to ID (rather than anemia due to other causes) and child mortality is difficult to characterize. Longitudinal studies without an intervention component that report death seldom incorporate specific biomarkers of iron status, such as serum iron, ferritin or transferrin receptor. Consequently, until additional data are available, we must estimate the contribution of anemia to mortality, under the assumption that low Hb concentrations in children reflect an iron deficit in approximately half of cases.

In a previous systematic review by Brabin *et al.* [12], little relation was found between increasing Hb concentration and decreasing case fatality rate in children. A higher risk of death was found in children with extremely low Hb (<50 g/L) relative to those with higher Hb, but a relation above this extreme cutoff was not found. In a sub-analysis of a single infant cohort from Malawi [12], the authors found a positive relation between Hb concentrations at six months of age and survival over the following six months. Stoltzfus *et al.* conducted a comprehensive analysis of the contribution of anemia to mortality in neonates and adult females, but concluded that the data available at the time were too highly confounded to describe this relation in children [6]. In an effort to fill this gap, our goal for the current meta-analysis was to incorporate studies since the Brabin *et al.* review [12] and to estimate the risk of death as a function of Hb in children aged one month to 12 years.

## 2. Experimental Section

### 2.1. Search Strategy

Electronic searches using four major databases—PubMed, Web of Science, EMBASE and Scopus—were performed in January 2014, to identify papers that might fulfill the inclusion criteria and were published since 2000 in order to cover the 13-year period since the Brabin *et al.* analysis [12]. The WHO regional databases were also searched, but did not yield any relevant results. The searches identified studies including the terms: (“hemoglobin” (Hb) OR “hematocrit” (Hct) OR “packed cell volume” (PCV)) AND (“child” OR “infant” OR “newborn” OR “preschool child”) AND (“mortality” OR “death” OR “fatality”). Term variations, including abbreviations (e.g., Hct), alternate spellings (e.g., haematocrit) and plurals (e.g., children) were also part of the search. Language was not a limiter in the search.

### 2.2. Inclusion/Exclusion Criteria

To be included in the final analysis, studies had to: (1) include children aged 28 days to 12 years; (2) contain at least two Hb/Hct/PCV categories; and (3) report the number of deaths in each Hb/Hct/PCV category. Non-human, intervention (as intervention could affect outcome) and review studies were excluded. In an effort to minimize the contribution of factors other than anemia to mortality, studies where all subjects had defined diseases and disorders—cancers, pneumococcal disease, inherited blood disorders (e.g., hemoglobinopathies), neurological disorders, renal disorders, splenic complications, cardiovascular disorders, sickle cell, diabetes, HIV, hemolytic disease—as well as those including birth and pregnancy complications, surgical intervention and emergency blood transfusion for all subjects were excluded to the greatest degree possible. However, as most studies occurred in hospital settings, it was virtually impossible to eliminate the contribution of all of these factors, which sometimes occurred in a subsample of the subjects.

### 2.3. Data Extraction Process

Independent data extraction was carried out by one reviewer, verified by a second reviewer and, in cases of disagreement, checked for accuracy by a third reviewer. Extracted data included the Hb or Hct category cutoff or mean concentrations, number of subjects and deaths in each Hb or Hct category, child age, where and when the study occurred and details pertaining to malaria, anemia etiology, iron biomarkers and comorbidities. The Newcastle-Ottawa Scale (NOS) [13] was used to assess the quality of the studies included in the meta-analysis. Each study was scored by the first author plus an independent reviewer, and any discrepancies were discussed before assigning the final score.

### 2.4. Analytic Procedures

PCV was divided by three to convert to Hb. We followed the method of Stoltzfus [6] by limiting the range of Hb in our analyses to 5–12 g/dL. Therefore, we set Hb categories reported as “<5 g/dL” to 5 g/dL and set categories reported as “>12 g/dL” equal to 12 g/dL. If a category was reported as “>5 g/dL”, we used 8.5 g/dL, the midpoint between this value and our upper meaningful cutoff, 12 g/dL. If a range was specified, the value was set as the midpoint of that specified range. Additionally, when Hb/Hct and death data were reported separately for disease groups, e.g., those who were transfused *vs.* not-transfused, parasitemic *vs.* aparasitemic, with *vs.* without malaria, we included only the “disease-free” group to minimize confounding of the relation between anemia caused by ID, the relation we were most interested in estimating, and mortality. The risk estimates of child mortality due to anemia were calculated using the LOGISTIC procedure in Statistical Analysis System (SAS) 9.3 (SAS Institute, Inc., Cary, NC, USA). Estimates are presented as the odds ratio of child mortality associated with a 1-g/dL increase in Hb and include 95% confidence intervals. The dependent variable in the logistic model was the ratio of deaths to the number of subjects in a given Hb category, *i.e.*, case fatality rate, and the independent variable was the Hb category. To estimate the overall effect across all studies, traditional meta-analysis was performed using the MIXED procedure. The individual study log odds ratios were specified as the dependent variable, with study variances included in a PARMS statement, following the method in SAS Users Group International paper 261-27 [14]. Separate models treating study as a fixed or as a random effect were fit; because the results did not differ, only results from the fixed model are presented.

In addition to the full 10-study meta-analysis, three sub-analyses were performed. First, we evaluated whether study quality as measured using the NOS scale affected our results and ran two additional models—one for only the low quality (≤6 points on the NOS) and another for only the high quality (>6 points on the NOS) studies. Second, for the two studies [15,16] that reported separate data for transfused and non-transfused children, we examined the individual case fatality rates for these groups in order to justify the exclusion of transfused subjects. These analyses were performed because blood transfusion is a potentially lifesaving intervention [17] that improves Hb and, thus, could modify the risk relation in which we were interested. Third, for the one study [18] that reported data for groups of children who were aparasitemic, parasitemic and parasitemic with malaria, we evaluated the risk relation separately for each of these groups. As anemia and malaria often coexist in developing regions and the presence of parasites and/or malaria contribute to morbidity and mortality, we felt it was important to estimate what effect these factors may have on our relation of interest. Lastly, we report the within-study relative risk of death across Hb categories (using the highest Hb group in each study as the referent group).

## 3. Results

### 3.1. Article Search and Selection

**Figure 1:**
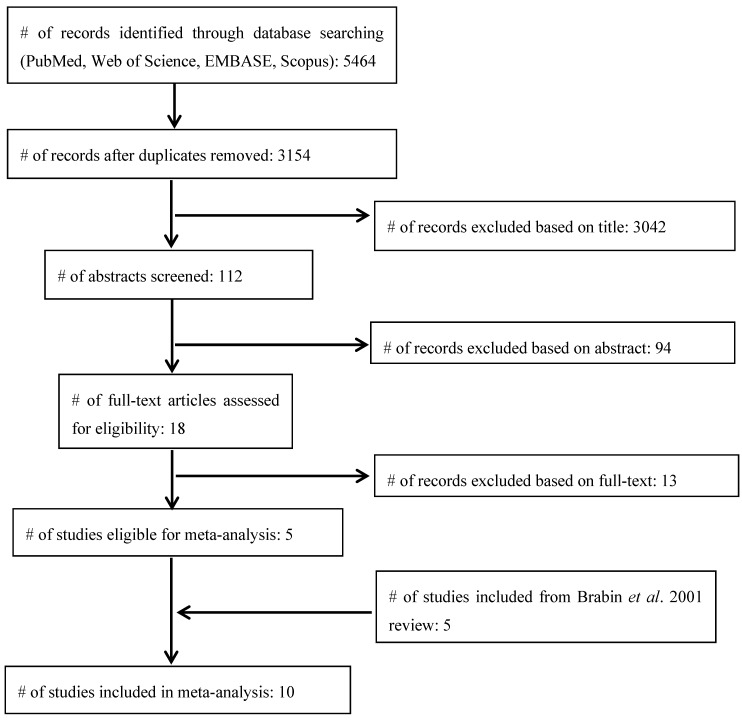
Article search and selection process.

A diagram illustrating the search and selection process is presented in Figure 1.

The number of articles retrieved from PubMed, Web of Science, EMBASE and Scopus was 1010, 1135, 1279 and 2040, respectively. Of the total 5464 articles, 2310 were duplicates, leaving 3154 articles to screen. Of these, 3042 were excluded based on title, leaving 112 abstracts to be reviewed. Ninety four papers were excluded based on the abstract, and the remaining 18 papers were read in full. Thirteen of the 18 papers could not be used because the authors did not report the number of deaths for each Hb/Hct/PCV category or the study design or subjects did not meet our criteria. One author [19] was contacted about missing data and responded to our request. This left five [19,20,21,22,23] new studies for our analysis that were added to the five [15,16,18,24,25] studies that were included in the previous analysis [12].

### 3.2. Reasons for Exclusion

The most frequent reasons for exclusion were as follows: subjects were outside the age range (*i.e.*, <28 days or >12 years of age), no measure of Hb/PCV/Hct was reported or death was not an outcome in the study. Additionally, many of the identified papers were ineligible based on design (e.g., intervention or cross-sectional studies), and a large number of studies included only subjects with sickle-cell disease, tuberculosis, hemoglobinopathies, cancer, HIV or other diseases or disorders. Some papers that seemed relevant (including children, measured Hb/Hct/PCV concentrations and reported cases of death) could not be used because the deaths were not categorized by Hb/Hct/PCV concentration and, thus, could not be modeled with logistic regression to predict the odds of death as a function of Hb concentration. One study [26] was excluded because the children included were part of a large vitamin A supplementation program, which may have affected the outcome. Another [27] only measured Hb concentration via cord blood, and as we felt that anemia status during the fetal period does not necessarily reflect anemia status during childhood (as was measured in the other included studies), we decided that this study did not fit in with our goal of estimating the burden of anemia during childhood on later mortality. Finally, we excluded eight studies that were included in the Brabin analysis for the following reasons: one [28] reported on the same sample as a study [18] that was already included, one [29] did not report the data clearly and no contact information was available for the author to clarify, one [30] only included children who received blood transfusions with no non-transfused group for comparison and five [31,32,33,34,35] did not categorize by Hb/Hct/PCV.

### 3.3. Studies Included

Characteristics of the included studies are presented in Table 1 and Table 2. Ten studies from the following countries met our inclusion criteria: Gambia [19], Kenya [15,16,18,22], Malawi [23], Tanzania [20,25], Uganda [21] and Zambia [24]. The sample size ranged from 217 to 2,203, representing a total of 11,811 children. Children were aged from 10 to 50 months on average. Three studies [16,19,23] used a case-control design, and the others were cohort studies. Two studies [23,24] reported mean Hb concentrations, while the others reported Hb ranges, which were then converted to midpoint Hb concentrations for the regression model. One study [25] reported PCVs, which were divided by three to convert to Hb. Excluding this study did not significantly influence our findings. Between two and four Hb groups were compared for each study. Within studies, the risk of mortality in the lower Hb group(s) relative to the higher, referent Hb group ranged from 1.0 (equal risk) to 13.1 (Table 1).

The majority of the studies occurred in hospital settings and in malaria-endemic regions. It follows that most studies included children with comorbidities, ranging from dehydration to HIV (Table 2). One study involved children drawn from the community who attended normal well-child clinic visits [19]. Only two studies [18,23] included iron-specific biomarkers, such as serum iron, ferritin and transferrin receptor, but did not report mortality rates for different categories of ferritin, TfR, *etc.*, and only one study provided information on anemia etiology [18] (Table 2).

**Table 1:** Characteristics of the included studies.

Author, Year	Country	*n* ^a^	Mean Age, Months	Study Design	Hb ^b^ (g/dL)	Hb Used for Analysis ^c^ (g/dL)	CFR, % (n_deaths_/n_total_)	Relative Risk
Lackritz *et al.*, 1992 [15]	Kenya	2017	10 (median)	Cohort	3.9–5.0 (not-transfused)	5.0	13 (49/382)	1.5
					3.9–5.0 (transfused)	5.0	10 (16/159)	1.2
					>5.0	8.5	8 (136/1,635)	1.0 (Ref.)
Lackritz *et al.*, 1997 [16]	Kenya	419	14	Case-control	<5.0 (not-transfused)	5.0	41 (48/116)	2.1
					<5.0 (transfused)	5.0	21 (40/187)	1.1
					≥5.0	8.5	20 (59/303)	1.0 (Ref.)
Newton *et al.*, 1997 [18]	Kenya	1446	50	Cohort	≤5.0 (aparasitemic)	5.0	9 (11/121)	1.6
					>5.0 (aparasitemic)	8.5	6 (74/1,325)	1.0 (Ref.)
					≤5.0 (parasitemic)	5.0	8 (13/159)	2.3
					>5.0 (parasitemic)	8.5	4 (26/735)	1.0 (Ref)
					≤5.0 (parasitemic with malaria)	5.0	8 (11/141)	2.3
					>5.0 (parasitemic with malaria)	8.5	3 (18/535)	1.0 (Ref.)
Mabeza *et al.*, 1998 [24]	Zambia	291	33	Cohort	6.0 (mean)	6.0	18 (39/222)	2.4
					9.2 (mean)	9.2	7 (5/69)	1.0 (Ref.)
Schellenberg *et al.*, 1999 [25]	Tanzania	2203	13	Cohort	<5.0	5	6 (10/177)	2.5
					5.0–8.3	6.7	5 (8/164)	1.6
					>8.3	10.2	2 (28/1,239)	1.0 (Ref.)
Ghattas *et al.*, 2003 [19]	Gambia	777	28 day-15 year (mean not reported)	Case-control	<7.0	6.0	75 (36/48) ^d^	1.6
					7.0–9.9	8.9	51 (161/318) ^d^	1.1
					10.0–10.9	10.5	46 (89/193) ^d^	1.0
					11.0–12.9	12.0	48 (105/218) ^d^	1.0 (Ref.)
Reyburn *et al.*, 2005 [20]	Tanzania	2191	12	Cohort	<5.0	5.0	8 (90/1,064)	2.9
					5.0–8.0	6.5	3 (33/1,127)	1.0 (Ref.)
Bachou *et al.*, 2006 [21]	Uganda	217	21	Cohort	<5.0	5.0	29 (4/14)	1.3
					≥5.0	8.0	23 (46/203)	1.0 (Ref.)
Obonyo *et al.*, 2007 [22]	Kenya	1116	16	Cohort	≤5.0	5.0	12 (28/233)	2.2
					>5.0	8.5	6 (49/883)	1.0 (Ref.)
Phiri *et al.*, 2008 [23]	Malawi	1134	23	Case-control	3.6 (mean) (all transfused)	5.0	17 (65/377)	13.1
					9.6 (mean)	9.6	3 (10/377)	2
					9.9 (mean)	9.9	1 (5/380)	1.0 (Ref.)

Hb, hemoglobin; CFR, case fatality rate; Ref., referent group. ^a^ n, total number of children included in our analysis, not necessarily the total number of children included in the referenced study. We excluded children outside of our age range, with a range of comorbidities, *etc.* See the Experimental Section for full inclusion/exclusion criteria. ^b^ These are the Hb categories as they were reported in each study. ^c^ Point estimates of Hb used for our analysis; calculated by taking the midpoint of the Hb range; a lower limit of 5.0 g/dL and an upper limit of 12.0 g/dL were used, since concentrations outside these limits are rare among the general iron-deficient population. ^d^ Each case (death) was matched to a control (survivor); thus, the CFRs for this study are artificially high (*i.e.*, *n*_total_ was approximately two-times *n*_deaths_, thus CFRs around 50%).

**Table 2:** Additional characteristics of the included studies.

Author, Year	Unique Exclusion Criteria	Sample Description	Comorbidities Present	% with Malaria	Specific Iron Measures	Anemia Etiology Described
Lackritz *et al.*, 1992 [15]	None	Admitted to hospital from 1989–1990 ^a^	Pneumonia (31%), gastroenteritis dehydration (4%), congestive heart failure (4%), sickle-cell (2%), marasmic kwashiorkor (2%)	76	No	No
Lackritz *et al.*, 1997 [16]	None	Admitted to hospital in 1991 ^b^	Respiratory illness (63%), malnutrition (22%), bacteremia (12%), HIV (8%)	33	No	No
Newton *et al.*, 1997 [18]	None	(1) asymptomatic, from the community; (2) admitted to hospital for any cause; (3) admitted to hospital with severe anemia, from 1989–1991 ^a^	Fever, GI bleeding, marasmus, sickle cell (10%)	29	Yes; plasma iron, Ft, TfR	Yes
Mabeza *et al.*, 1998 [24]	Non-malarial causes of altered consciousness	With cerebral malaria, admitted to hospital from 1990–1994 ^b^	Not described	100	No	No
Schellenberg *et al.*, 1999 [25]	Abnormal CSF	With malaria, admitted to hospital from 1995–1996 ^a^	Splenomegaly, hepatomegaly, hypoglycemia, vomiting, respiratory distress, dehydration	100	No	No
Ghattas *et al.*, 2003 [19]	Deaths due to accidents	From community, attending regular well-child clinics at medical center from 1950–1997 ^b^	Malnutrition, gastroenteritis, other infections	Not reported	No	No
Reyburn *et al.* 2005 [20]	Anemia due to malignancy or trauma	Admitted to hospital in 2002 with intention to treat for malaria ^b^	Prostration, impaired consciousness, confusion, respiratory distress, jaundice	47	No	No
Bachou *et al.*, 2006 [21]	None	Severely malnourished, admitted to hospital in 2003 ^a^	Fluid overload, septicemia, pneumonia, tuberculous meningitis, hypothermia, hypoglycemia, hepatitis (1 case), cerebral malaria (1 case), measles (1 case)	47	No	No
Obonyo *et al.*, 2007 [22]	None	Admitted to hospital in 2002 ^a^	Pneumonia (29%), diarrhea (15%)	83	No	No
Phiri *et al.*, 2008 [23]	Length <49 cm	Admitted to hospital for severe anemia (cases), other illness (hospital controls) or community of each case (community controls); 2002–2006 ^b^	HIV (11%), edema, septicemia, pneumonia, tuberculous meningitis, drug reactions, hypothermia, hypoglycemia, hepatitis, cerebral malaria, measles	41-59	Yes; Ft, TfR	No

^a^ Low quality (≤6 points on the Newcastle Ottawa Scale); ^b^ high quality (>6 points on the Newcastle Ottawa Scale).

### 3.4. Logistic Regression Meta-Analysis

**Figure 2:**
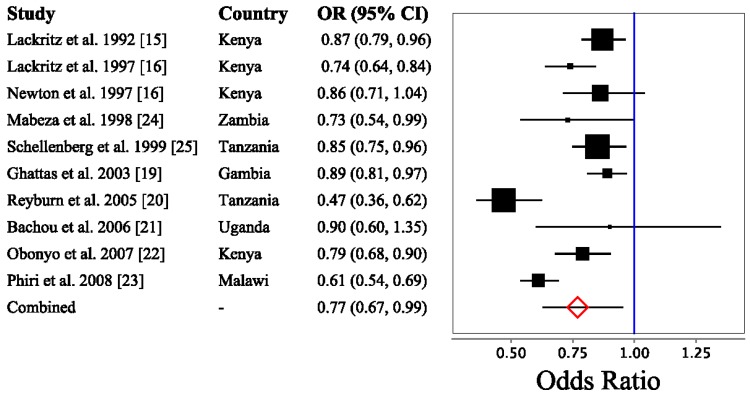
Estimated odds of death associated with a 1-g/dL increase in hemoglobin.

The results of the logistic regression and meta-analysis of the ten included studies are shown in Figure 2. The combined estimate of 0.76 (95% CI 0.62–0.93) reveals that for each 1-g/dL Hb increase, the risk of child mortality decreases by 24% (95% CI 7%–38%). Individual study odds ratios ranged from 0.47 to 0.90, with the upper 95% confidence limit exceeding 1.0 in only two studies [18,21].

### 3.5. Sub-Analyses of the Effects of Study Quality, Transfusion and Malaria Parasitemia on the Relation between Hb and Mortality

Five [15,18,21,22,25] of the included studies scored ≤6 out of a possible nine points on the NOS [13] (*i.e.*, low quality), while five [16,19,20,23,24] scored >6 (*i.e.*, high quality). Separate sub-analyses of low and high quality studies revealed different associations for each set of studies. In the set of studies ranked as having low quality, we estimated a non-significant combined OR of 0.87 (95% CI 0.62–1.21), whereas in the set of studies ranked as having high quality, the estimated OR was significant at 0.63 (95% CI 0.48–0.83).

Two studies [15,16] presented data separately for transfused and non-transfused children. We compared the Hb <5.0 g/dL (non-transfused) and Hb <5.0 g/dL (transfused) groups in order to confirm that transfusion benefits survival and, thus, would confound the relation in which we were interested. As expected, transfusion had a beneficial effect on survival, with lower case-fatality rates in the transfused *versus* non-transfused groups (Table 1).

One study [18] presented data separately for children who were aparasitemic, parasitemic or parasitemic with malaria. The risk of dying between children with lower *versus* higher Hb was slightly lower in the aparasitemic group compared to the other groups (Table 1). The OR for the aparasitemic group of 0.86 (95% CI 0.71–1.04), though not statistically significant, was slightly higher than the others: 0.78 (95% CI 0.64–0.95) and 0.78 (95% CI 0.62–0.97), respectively, for the parasitemic and parasitemic with malaria groups. However, given that these are data from a single site, interpretations should be cautious.

## 4. Discussion

### 4.1. Main Finding and Implications

This systematic review provides up-to-date coverage of published studies examining the relation between anemia during childhood and subsequent death, incorporating information on nearly 12,000 children from six African countries. Our pooled finding revealed that, for each unit increase in Hb, the risk of child death falls by 24% (95% CI 7%–38%). This finding has far-reaching implications regarding the prevention of child mortality. In 2012, there were 48 deaths per 1000 live births among children under five (both anemic and non-anemic), with 44 percent of these occurring in the first 28 days of life [3]. Two hundred and seventy three million children under five are estimated to be anemic (Hb <11 g/dL), with 3.7% or 10 million of these considered to be severely anemic (Hb <7 g/dL) [7]. After subtracting the number with severe anemia, we then applied our finding of a 24% reduction in mortality risk and estimate that around 1.8 million deaths could be avoided each year if Hb were to increase by 1 g/dL among anemic children aged 28 days to 5 years. This estimate is most likely conservative given that the global U5MR may be higher than 48 per 1000 live births if only anemic children are considered. Importantly, anemia can be a direct (when severe) or indirect (when mild or moderate) contributor to death; however, given the low prevalence of severe anemia (3.7% of anemia cases in children <5 years [7]) in contrast to mild and moderate anemia (96.3% of anemia cases in children <5 years [7]), the vast majority of anemia-related deaths are related to mild and moderate anemia. Because of the graded risk relation demonstrated here, even a modest improvement in Hb concentration could reduce mortality rates in infants and young children.

### 4.2. Iron Interventions in Malaria-Endemic Regions

Iron supplementation is the most commonly-used strategy to increase Hb concentration and reduce anemia. Because of the high prevalence of both ID and anemia among children in developing countries, universal supplementation of children beginning at six months of age was recommended [36,37]. However, the safety of universal iron supplementation in malaria-endemic regions has been questioned. In 2003, a large study in Zanzibar found an increased risk of malaria-associated morbidity and mortality in iron-sufficient children receiving iron supplements [38]. This shifted the balance of evidence on safety, because a prior summary of placebo-controlled randomized iron supplementation trials conducted in malaria-endemic populations with iron deficiency anemia (IDA) had shown that iron supplementation resulted in a Hb increase of 1.25 g/dL (95% CI 1.20–1.30) and did not significantly increase the risk of a clinical malaria attack [37]. Subsequent studies have evaluated the efficacy of alternate iron delivery mechanisms in malaria-endemic areas. Overall, a systematic review of 55 RCTs with varying dose and duration found a pooled estimate for change in Hb concentration of 0.74 g/dL with iron supplementation, with greater responses in those with lower baseline Hb [39]. These findings underscore the point that improving Hb by 1 g/dL is possible and can be accomplished with the use of relatively simple iron supplements among moderately anemic children (Hb 7–11 g/dL) aged 28 days to 5 years. In response to the findings in Zanzibar, a joint statement from the WHO and UNICEF was issued in 2006 recommending that iron supplementation only be given to children who are anemic or at risk for ID [40], bringing into question universal iron supplementation programs in malaria-endemic regions. Our finding of no difference in ORs between groups with and without malaria in the study by Newton *et al.* [18] support prior evidence that improving Hb reduces mortality risk similarly in children with malaria and in those without malaria. More recent work has provided convincing evidence that iron intervention via a micronutrient powder (MNP) in these areas is safe when malaria prevention strategies, such as insecticide-treated bed nets and malaria prophylaxis, are available [41]. This double-blind cluster randomized trial was conducted in Ghana with approximately 2,000 children aged 6–35 months. The group of infants receiving daily iron-containing MNP for five months had a lower malaria incidence compared to those receiving MNP without iron (RR 0.87; 95% CI 0.79–0.97). Sub-analyses revealed that malaria incidence was lower in those with moderate anemia (Hb of 7.0–10.0 g/dL) in the iron-MNP group *vs.* the MNP without iron group (RR 0.74; 95% CI 0.65–0.86), but this relation did not hold for those with Hb >10.0 g/dL (RR 0.96; 95% CI 0.84–1.12). In sum, the most recent evidence suggests that, when appropriate malaria control strategies are in use, iron intervention via MNP is both a safe and effective tool to reduce ID and IDA in areas where malaria is intense and may be particularly effective in children with relatively lower Hb concentrations. However, it is well understood that a multifaceted approach is required to fully eliminate anemia. A case-control study [42] in Malawian children investigated the etiology of anemia and found that iron deficiency was not more prevalent in hospitalized children with anemia compared to non-anemic community controls. Subsequent analysis [43] indicated that the most common etiological factors were vitamin A deficiency, malaria, iron deficiency and vitamin B12 deficiency, which were present in 92.3, 59.5, 46.6 and 30.4 percent of the 234 children with severe anemia included in their analysis. This finding understates the fact that iron intervention alone is unlikely to resolve all cases of anemia.

### 4.3. Comparison with Other Studies

Other studies that did not report deaths by Hb category, and thus did not meet the criteria for inclusion in the current analyses, are in general agreement with our findings. Brabin and colleagues [12] estimated a hazards ratio of 0.58 (95% CI 0.38–0.89) in a community cohort of 216 Malawian infants with individual Hb concentrations ranging from 6–10 g/dL at six months of age and who did not receive iron supplementation before six months, indicating that an Hb decrease of 1 g/dL was associated with a 1.72-times higher risk of death during the following six months. In a review [44] of Taiwanese children without underlying diseases admitted to a hospital over a 10-year period, a lower Hb concentration was independently associated with a higher risk of dying. A cohort of HIV-uninfected children (*n* = 419) in Zambia [45] followed this same trend; compared to children with Hb >8.0 g/dL at 2 to 8 months of age, those with lower Hb concentration were 1.6-times more likely to die before the age of three. These risk estimates are in line with the relative risks of death found for the studies included in our meta-analysis (Table 1).

### 4.4. Strengths and Limitations

We performed an exhaustive literature search of four major databases and a careful screening process to select appropriate articles best suited to answer our research question. We used rigorous selection criteria and tried to exclude children with comorbid health conditions that might bias our risk relation estimates. Additionally, our estimates may be conservative, because we focused on Hb concentrations within the 5–12 g/dL range and assigned midpoint Hb values to the groups rather than model Hb as a continuous variable, which was not an option, given the available data. Looking more closely, the actual mean Hb for a group reported as “<5 g/dL” was often between 3–4 g/dL, and the actual mean Hb for a group “>5 g/dL” was often between 5–6 g/dL, as many of the included studies involved patients admitted to a hospital for severe anemia. By converting these “<5 g/dL” and “>5 g/dL” groups to 5 g/dL and 8.5 g/dL, respectively, for use in our regression model, the Hb group difference widened (a two-unit difference between groups became a 3.5-unit difference). Thus, as an artifact of a wider Hb group difference in the model, the estimate of death per unit increase becomes smaller (more conservative), so it is likely that our finding of a 24% reduction in mortality per unit increase in Hb concentration is an underestimate of the true risk relation.

All studies in our analyses occurred in Africa, and all but one study [23] (which included a community control group) occurred exclusively in hospital settings, where health burdens, such as anemia, malnutrition, infection, respiratory distress and numerous other conditions co-exist and where healthcare is available. No studies in our analyses occurred in non-malaria areas. Therefore, we cannot say with certainty that our findings apply outside of Africa, in non-hospital settings or in non-malaria areas. Though these contextual limitations preclude the generalization of our findings, our sub-analyses of children who were transfused *vs.* non-transfused and of children who were aparasitemic *vs.* parasitemic *vs.* parasitemic with malaria, albeit based on the data from single sites, shed some light on the generalizability of the risk relation. Our finding of a higher risk of death among children who were not transfused *vs.* transfused confirms that transfusion benefits survival. However, as transfusion for anemia is typically reserved for the most severe cases and involves the risk of unintentional infection in low-resource contexts [46], alternate strategies, such as iron intervention are warranted to improve Hb concentrations in cases of mild or moderate anemia. Additionally, we found equal ORs for groups with and without malaria (discussed further below). Given that anemia is a global problem occurring in both developing and developed countries and affecting children in all types of settings [8]—community, non-hospital, without malaria, *etc.*—future studies on anemia and child mortality across multiple regions and contexts are needed.

### 4.5. The Contribution of Malaria to Anemia-Related Child Mortality

Malaria is a known cause of anemia. In their report [6] of the contribution of anemia to perinatal mortality, Stoltzfus *et al.* performed a subgroup analysis comparing the OR in *P. falciparum* endemic *vs.* not endemic regions and found that the (maternal and perinatal) mortality risk relation with anemia was greater in malaria-endemic regions. In one study [18] reporting case fatality rate separately for parasitemic *vs.* parasitemic with malaria groups, we found no difference in the risk relation in parasitemic children with or without malaria. In all other studies, the percentage of children with malaria (Table 2) was stated for the full sample (not for those who died *vs.* survived or by Hb group); therefore, a multi-study model examining the effect of malaria on the relation between Hb and death was not possible. Given that ID and malaria both disproportionately affect children across Africa and southern Asia, the effects of these afflictions are difficult to disentangle.

### 4.6. Recommendations for Future Reports

One concerning observation was that newer and older studies were ranked similarly on the NOS scale in terms of study quality. We have several suggestions regarding study design and reporting strategy for researchers in this field. For cohort studies, it is important that the sample is representative of the exposed individuals in the community; many of the studies included in our analyses included only children admitted to a hospital. It is also important to have a sufficiently long follow up period for the outcome of interest to occur.

Another issue brought to our attention during the article screening process was the lack of a standardized reporting strategy. A number of studies could not be included in our analysis due to the aggregation of data in the report, e.g., reporting total deaths instead of deaths classified by Hb concentration. We suggest that future reports present data in a disaggregated fashion. Most studies either did not investigate or omitted details pertaining to anemia etiology and the specific cause of death. Given the overlapping comorbidities in the majority of children who die in developing countries, there are likely several contributors to child mortality, and understanding how anemia specifically contributes to death is important. Omission of these specifics is costly in that: (1) the usefulness of the report to a wide audience with varying interests is lessened; and (2) the development of targeted public health strategies is precluded, given that such strategies depend upon a detailed understanding of the underlying health factors contributing to the event. Regarding ID, the true contribution of ID to child mortality will remain unknown until specific biomarkers of iron status are used on a wider scale, particularly in hospital settings as part of routine testing. The fact that we found only two studies assessing iron-specific biomarkers illustrates this problem. Additionally, when assessing iron status, inflammatory state should always be measured and reported due to the confounding nature of inflammation on indicators of iron status, such as ferritin and transferrin [47].

## 5. Conclusions

Identifying and quantifying the problem—in this case, that low Hb concentration in children is related to a higher likelihood of later death—is only the first step toward public health improvement and must be coupled with strategies to effectively reduce the burden of disease. Though not the only strategy to combat anemia, iron intervention in anemic children can improve Hb concentrations [37,39,48], supporting the feasibility of shifting Hb by 1 g/dL to reduce mortality. Importantly, the design of effective and safe interventions to deliver iron in malaria-endemic areas requires further investigation and resolution [49,50]. It is critical to routinely measure and report specific iron status indicators, such as concentrations of ferritin and transferrin receptor, along with those of inflammatory markers, such as C-reactive protein and alpha-1-acid glycoprotein, in order to truly understand the contribution of ID and IDA to child mortality. With an improved understanding of the underlying etiology behind anemia, including ID, policy makers can formulate effective public health strategies to reduce the large burden of childhood anemia worldwide.
